# Wee1 Inhibitor AZD1775 Effectively Inhibits the Malignant Phenotypes of Esophageal Squamous Cell Carcinoma *In Vitro* and *In Vivo*

**DOI:** 10.3389/fphar.2019.00864

**Published:** 2019-08-02

**Authors:** Shuning Bi, Qiuren Wei, Zhijun Zhao, Liang Chen, Chaojie Wang, Songqiang Xie

**Affiliations:** ^1^Institute of Chemical Biology, College of Pharmacy, Henan University, Kaifeng, China; ^2^Department of Medicine and Therapeutics, Luohe Medical College, Luohe, China; ^3^The Key Laboratory of Natural Medicine and Immuno-Engineering, Henan University, Kaifeng, China

**Keywords:** ESCC, Wee1, AZD1775, apoptosis, metastasis

## Abstract

Esophageal squamous cell carcinoma (ESCC) is a common malignant diagnosed cancer with increasing incidence rate and few treatment options. As a specific small-molecule inhibitor of the Wee1 tyrosine kinase, AZD1775 has previously shown potent antitumor effect on multiple types of cancer in various preclinical studies and clinical trials. However, the expression of Wee1 and the role of AZD1775 in ESCC remain unclear. In the present study, we found that the expression of Wee1 was much higher in ESCC cell lines and clinical samples than that of the corresponding controls. In addition, we demonstrated that AZD1775 exhibited strong inhibitory effect against Wee1 kinase in both tested ESCC cells at nanomolar concentrations. Moreover, AZD1775 effectively suppressed ESCC cell growth and triggered apoptosis *via* the mitochondrial-dependent signaling pathway. AZD1775 also diminished cell migration and invasion as well as the expression of MMP-2 and MMP-9. Interestingly, knockdown of Wee1 displayed a similar inhibitory effect of AZD1775 on ESCC cells. In addition, there was a synergism between AZD1775 and 5-fluorouracil or cisplatin in inducing cell death. More importantly, the *in vivo* experiments also demonstrated that AZD1775 potently inhibited ESCC cell growth and metastasis. In summary, our data suggest that the Wee1 inhibitor AZD1775 may be a potential therapeutic agent and warrants a clinical trial for patients with ESCC, even those with metastasis.

## Introduction

Esophageal cancer, a tumor of the digestive tract, is the eighth most prevalent types of cancer and ranks as the sixth leading cause of cancer-associated death worldwide (McGuire, 2016; Lin et al., 2018; Li et al., 2019). Esophageal squamous cell carcinoma (ESCC), as the most predominant histological form of the disease (>90%), is particularly prevalent in China (Torre et al., 2015; Fung et al., 2016). Although novel surgical techniques in combination with various adjuvant treatment modalities, including chemotherapy and radiotherapy, have been used, the overall 5-year survival rate of ESCC patients is still <20% (Enzinger and Mayer, 2003; Lin et al., 2018), mainly due to late diagnosis, metastasis, and frequent tumor recurrence (Li et al., 2009a; Lin et al., 2018). Therefore, there is an urgent need to find novel therapeutic approaches to improve the outcomes for these patients with ESCC.

Wee1 is a tyrosine kinase that plays a crucial role in halting the G_2_/M cell cycle checkpoint for DNA repair (Matheson et al., 2016; Geenen and Schellens, 2017). In the presence of cellular DNA damage, Wee1 phosphorylates CDK1 at Y15 residue, thus preventing the Cyclin B-CDK1 complex from driving cells into mitosis, leading to cell cycle arrest in G_2_ phase, allowing time for DNA repair (Russell and Nurse, 1987; Harris et al., 2014; Matheson et al., 2016; Geenen and Schellens, 2017). High expression of Wee1 has been found in several cancer types including acute leukemia (Weisberg et al., 2015), rhabdomyosarcoma (Stewart et al., 2018), glioblastoma (Lescarbeau et al., 2016), lung cancer (Hai et al., 2017; Richer et al., 2017), and head and neck squamous cell carcinoma (Tanaka et al., 2015). Importantly, high expression of Wee1 has been associated with tumor metastasis and poor prognosis (Ge et al., 2017; Yuan et al., 2018). These reports suggest that Wee1 may play a crucial role in tumorigenesis and progression. Indeed, overexpression of Wee1 significantly promotes the proliferation, migration, and invasion in gastric cancer cells; Vice versa, RNA interference (RNAi)-mediated knockdown of Wee1 dramatically suppresses these effects (Kim et al., 2016). Therefore, Wee1 has been thought as a promising therapeutic target for human cancers.

AZD1775 is an orally available, highly specific ATP-competitive small-molecule inhibitor for Wee1 tyrosine kinase activity. AZD1775 exerts antiproliferative effect and induces apoptosis in acute lymphoblastic leukemia cells, lung cancer cells, colorectal cancer cells, and laryngeal squamous cell carcinoma cells and in human xenografts in nude mice (Chen et al., 2017; Richer et al., 2017; Webster et al., 2017; Duan et al., 2018; Yuan et al., 2018). Of note, AZD1775 has been reported to potentiate the antitumor efficacies by DNA-damaging agents including gemcitabine, carboplatin, or cisplatin at tolerated doses in nude rats (Hirai et al., 2009). In addition, AZD1775 displays a synergistic antitumor activity when combined with Sirt1 inhibitor Ex527 in lung cancer xenograft model *in vivo* (Chen et al., 2017). A phase I study about Wee1 inhibitor AZD1775 alone or in combination with gemcitabine, cisplatin (CDDP), or carboplatin in patients with advanced solid tumors showed that AZD1775 was tolerable and safe as a single agent or in combination with chemotherapy at doses associated with target engagement (Do et al., 2015; Leijen et al., 2016a). Encouragingly, a phase II study also provided clinical proof that AZD1775 could enhance carboplatin efficacy in patients with TP53-mutated ovarian cancer refractory or resistant to first-line platinum-based therapy within 3 months (Leijen et al., 2016b). Whether AZD1775 is active against ESCC has not been reported previously.

## Materials and Methods

### Chemicals and Antibodies

AZD1775 was purchased from Selleck Chemicals (Shanghai, China). CDDP and 5-FU were purchased from Sigma-Aldrich (Shanghai, China). Antibodies against Wee1, CDK1, phospho-CDK1 (Y15), histone H3, phospho-histone H3 (S10), H2A.X, γH2A.X, MMP-2, MMP-9, PARP, caspase-3, active caspase-3, Bax, Bcl-xL, XIAP, survivin, cytochrome c, AIF, and COX IV were obtained from Cell Signaling Technology (Beverly, MA); anti-Ki67 antibody was obtained from Abcam (Cambridge, UK). Antibody against actin was purchased from Sigma-Aldrich (Shanghai, China). Antirabbit immunoglobulin G and antimouse immunoglobulin G horseradish peroxidase (HRP)-conjugated secondary antibodies were from ZSBG-Bio (Beijing, China).

### Patients and Specimens

A total 63 pairs of ESCC and the corresponding normal tissues were obtained from the First Affiliated Hospital of Henan University between 2015 and 2018. All patients enrolled in the research had not received chemotherapy or radiation treatment. Patients’ age ranged from 50 to 85 years at the point of surgery. The research was approved by the ethics committee of the First Affiliated Hospital of Henan University. Written informed consents were obtained from all patients before the study.

### Cell Culture

Hunan ESCC cells EC109 and KYSE150 were obtained from the Cell Bank of the Chinese Academy of Sciences (Shanghai, China) and cultured in RPMI 1640 medium (Invitrogen) containing 10% (*v/v*) fetal bovine serum (FBS) at 37°C in a humidified incubator containing 5% CO_2_. The immortalized human esophageal epithelial cell Het-1A was obtained from the American Tissue Culture Collection (ATCC, Manassas, VA, USA) and cultured in DMEM culture medium supplemented with 10% FBS. The cells were recently tested for mycoplasma contamination.

### MTT Assay

Cells were seeded in 96-well flat-bottom plates (Corning) at a density of 3 × 10^3^ cells per well and treated with increasing concentrations of AZD1775 for 3 days. Twenty microliters of MTT solution (5 mg/ml) was added 4 h before reading the absorbance at a wavelength of 570 nm. Control cells received <0.1% DMSO containing medium. The drug concentration resulting in 50% inhibition of cell growth (IC_50_) was determined using the GraphPad Prism version 7.0 (GraphPad Software, San Diego, CA).

The synergism of combinational drugs was assessed by the median-effect method of Chou and Talalay (Chou, 2010). The effects of combinations were estimated using the CalcuSyn software. The combination index (CI) was the ratio of the combination dose to the sum of the single-agent doses at an isoeffective level. Therefore, CI > 1, antagonism; CI = 1, additive; and CI < 1 indicates synergy.

### Colony Formation Assay

The colony formation assay was performed as previously described (Li et al., 2014b). Briefly, ESCC cells were treated with the increasing concentrations of AZD1775 for 48 h, cells were then harvested, and a total of 500 cells per well were plated in six-well plates and cultured for 2 weeks until visible colonies formed. Surviving colonies were fixed and stained with 0.1% crystal violet for 20 min at room temperature. Microscopic colonies consisted of more than 50 cells were counted.

### Soft Agar Clonogenic Assay

For anchorage independence, a two-layer soft agar-containing media was plated in 24-well flat-bottom plates as described previously (Chen and Pan, 2017). Briefly, the lower layer (300 μl) was DMEM growth medium containing 10% FBS and 0.8% agar. The upper layer contained 2,000 ESCC cells (pretreated with various concentrations of agents for 48 h) in RPMI 1640 medium containing 10% FBS and 0.4% agar. After incubation 14 days in a humidified incubator, the colonies composed of more than 50 cells were counted using an inverted phase-contrast microscope as previously described (Chen and Pan, 2017).

### Apoptosis Assay

For measuring apoptosis, ESCC cells were treated with the indicated concentrations of AZD1775 for 48 h. Cells were collected and resuspended in 1× binding buffer. Annexin V-FITC was then added and incubated at room temperature for 15 min in the dark. The cells were washed with 1× binding buffer; after the PI solution was added to the cell suspension, apoptosis was immediately assessed using the BD FACSVerse flow cytometer and its software.

### Western Blotting Analysis

Western blotting analysis was performed using the whole cell lysates prepared in RIPA buffer (Chen and Pan, 2017). For detection of cytochrome c and AIF, cytosolic fraction was prepared in digitonin extraction buffer (Chen and Pan, 2017). Protease inhibitor cocktail (Roche, Indianapolis, IN), 10 mM β-glycerophosphate, 1 mM sodium orthovanadate, 10 mM NaF, and 1 mM phenylmethylsulfonyl ﬂuoride were added to the buffer mentioned above. Equal amounts of total proteins were separated using 10–15% non gradient SDS-PAGE and then transferred onto nitrocellulose membranes. After blocking with 5% dried skimmed milk, the membranes were then incubated with the primary antibodies overnight. After probing with appropriate secondary antibodies overnight, the corresponding horseradish peroxidase (HRP)-conjugated secondary antibodies were used against each primary antibody. Protein bands were detected with the enhanced chemiluminescence (ECL) detection reagent (Beyotime, Shanghai, China).

### RNA Extraction and Quantitative Real-Time PCR (qRT-PCR)

Total mRNAs were isolated using the TRIzol reagent (Invitrogen, Shanghai, China) according to the manufacturer’s manuals. Reverse transcription was performed using PrimeScript RT Master Mix (TaKaRa, Dalian, China). The qRT-PCR was carried out using SYBR Premix Ex Taq II (TaKaRa, Dalian, China) on the ABI Prism 7, 900 System (Thermo Fisher Scientific). The cycle threshold values did not differ by more than 0.5 among the triplicates. Relative expression differences were calculated using the 2^−ΔΔCt^ method. GAPDH gene was used as internal control. Primer sequences are listed in **Supplementary Table S1**.

### Cell Cycle Analysis

For cell cycle analysis, ESCC cells were treated with or without nocodazole (Sigma-Aldrich, 10 ng/ml) for 8 h, followed by 250 nM AZD1775 treatment for 16 h (Bukhari et al., 2019). Cells were harvested, washed with PBS three times, and fixed in 70% chilled ethanol for at least 24 h at −20 C. Propidium iodide (Sigma-Aldrich, 50 μg/ml) solution containing RNAse A (Sigma-Aldrich, 10 μg/ml) was then added and incubated for 1 h at 37 C in the dark. Samples were analyzed using a BD FACSVerse flow cytometer and its software.

### Measurement of Mitochondrial Transmembrane Potential

ESCC cells were exposed to 0.5 μM AZD1775 for different durations. Cells were collected and stained with MitoTracker probes (MTGreen and CMXRos, Eugene, OR, USA) according to the manufacturer’s instructions. The cells were then subjected to flow cytometry analysis for the mitochondrial transmembrane potential as described previously (Chen and Pan, 2017).

### Wound Scratch Assay

For wound scratch assay, KYSE150 and EC109 cells were platted in six-well flat-bottom plates (Corning) and grown to a confluence cell monolayer. After scratching with a 200-μl sterile pipette tip, the wells were washed with 1 × PBS to remove the floating cells. The cells were cultured in RPMI 1640 medium with or without AZD1775 and photographed under an inverted microscope at different time points after drug treatment. The percentage of wound closure was assessed as (original gap distance − gap distance at 24 h or 48h)/original gap distance × 100%.

### Transwell Assays

The transwell assays were performed in transwell chambers (8-μm pore size, Costar) according to the manufacturer’s instructions. In brief, after treatment with or without AZD1775 for 48 h, cells were harvested and resuspended in FBS-free RPMI 1640 medium. For transwell migration assay, 200 μl of serum-free medium (containing 5 × 10^4^ cells) was placed into the upper chamber of each insert, while the lower compartment had 600 μl of RIMI 1640 medium with 20% FBS. After incubation for 24 h at 37 °C, the remaining tumor cells inside the upper chamber were removed with cotton swabs. The cells on the lower surface of the membrane were fixed with paraformaldehyde, stained with 0.1% crystal violet, and counted under a light microscope. The invasion assay was performed using a same procedure, except that the inserts were coated with Matrigel, and then 1 × 10^5^ ESCC cells were added to the upper chamber.

### Immunofluorescence Staining

The immunofluorescence staining was performed as previously described (Chen et al., 2013). Briefly, after treatment with AZD1775, KYSE150 and EC109 cells were washed with ice-cold PBS three times and fixed in 4.0% paraformaldehyde. After permeabilization with 0.1% Triton X-100, the cells were blocked of nonspecific binding by incubation with 5% bovine serum albumin (BSA) in PBS and then incubated with primary anti-p-CDK1 (Y15), anti-p-HH3 (S10), and anti-γH2A.X overnight at 4°C. The staining was completed with FITC-labeled goat antimouse and Alexa Fluor^®^ 594 goat antirabbit second antibody (1:500, ZSBG-Bio, Beijing, China). After incubation for 1 h at room temperature, the cellular nuclei were counterstained with Hoechst33342 (Sigma, USA) for 15 min at room temperature in the dark. All photographs were taken at a magnification of 200× using high content screening (HCS) (Thermo Scientific Cellomics ArrayScan Vti, Cellomics, Inc., Pittsburgh, PA).

### Lentiviruses Production and Infection of Target Cells

Specific shRNAs targeting Wee1 and a scramble shRNA (pLKO.1-puro-nontarget shRNA, shNC) were purchased from Sigma-Aldrich. The indicated sequences were described in **Supplementary Table S2**. To make lentiviruses, the scramble or shRNA constructs targeting Wee1, envelope plasmid (pMD2.G-VSV-G), and packaging plasmid (pCMV-dR8.91) were transfected into 293T cells using the Lipofectamine 2000 reagent (Invitrogen, Thermo Fisher Scientific, Inc). according to the manufacturer’s instruction. After 48 h incubation period, the virus containing supernatants were harvested, concentrated, and used to infect ESCC cells in the presence of 8 μg/ml polybrene (Sigma-Aldrich). Stable cell lines expressing Wee1 shRNA (shWee1#1 and shWee1#2) were selected in the presence of 1.5 μg/ml puromycin (Sigma-Aldrich) for 4 weeks. Western blotting analysis was used to determined the efficiency of knockdown.

### Mouse Models of Tumorigenesis and Metastasis

Male BALB/c nude mice (5- to 6-week-old, 18–20 g) were purchased from Beijing Vital River Laboratory Animal Technology Co (Beijing, China). All mice were bred and maintained in barrier facilities of Henan University with controlled humidity (40–50%), temperature (20 ± 2 °C), and on a 12-h light/dark cycle. Mice were housed in isolator cages (four mice per cage). During the experimental period, the standard pellet food and water were provided *ad libitum*. All experimental procedures were approved by the Institutional Animal Care and Use Committee of Henan University.

For *in vivo* tumorigenesis experiment, KYSE150 cells (5 × 10^6^ cells in PBS suspension) were subcutaneously implanted into the left dorsal flank of each mouse (Li et al., 2009b; Li et al., 2014a). Tumor growth were measured with calipers every other day, and volume was calculated by the following formula: tumor volume = *length* × *width* ^2^ × 0.5. When the tumor volumes reached ∼100 mm^3^ in size, the mice were randomly split into two groups (*n* = 8 per group). Mice were administrated daily with MK-1775 (60 mg/kg) or vehicle (0.5% methylcellulose) *via* oral gavage for 2 weeks. Mice were then anesthetized with isoflurane before being killed by cervical dislocation. Tumors were immediately removed, weighed, fixed, or kept at −80°C. The body weight, feeding behavior, and motor activity of each animal were monitored every day as indicators of general health.

For the lung metastasis assay, the BALB/c nude mice were intravenously injected with KYSE150 cells (1 × 10^6^ in 100 μl PBS) *via* lateral tail veins (Chen and Pan, 2017). Twenty-four hours later, the mice were randomly divided into two groups (*n* = 8 per group) and were administrated daily with AZD1775 (60 mg/kg) or vehicle (0.5% methylcellulose) by gavage for 2 weeks. Six weeks later, all of the mice were killed by cervical dislocation; the lungs were fixed in Bouin’s solution (25% formaldehyde, 5.0% acetic acid, and 75% saturated picric acid) and embedded in paraffin using the routine method. The metastatic colonies on the lung surface in each mouse were counted, and H&E staining was used to detect the histological evidence of the tumor phenotype within the lungs.

### Immunohistochemistry and Hematoxylin and Eosin Staining

The formaldehyde-fixed tissues were dehydrated and embedded in paraffin according to the routine method. The paraffin-embedded tissues were then cut into pieces (4 μm) and placed on polylysine-coated slides. For immunohistochemistry (IHC) staining, the procedure was performed as previously described in our previous studies (Chen and Pan, 2017). Briefly, the tissue slides were deparaffinized and rehydrated. After treatment with 3% H_2_O_2_ for 10 min, the tissue slides were retrieved for 15 min in 10 mM citrate (pH 6.0) using a microwave oven. After 20 min of preincubation in 5% bovine serum albumin to prevent nonspecific staining, the slides were incubated overnight using primary antibodies against Ki67 (1:100), anti-p-CDK1 (1:200), p-HH3 (1:100), and γH2A.X (1:100) in a humidified container at 4°C. Staining results were visualized by sequential incubations of slides with an EnVision + System-HRP (DAB; DAKO) used according to manufacturer’s manuals, followed by hematoxylin counterstaining. For hematoxylin and eosin staining (H&E staining), the paraffin-embedded tissue sections were deparaffinized in xylene and rehydrated with graded ethanol. Subsequently, the slides were stained with H&E using Hematoxylin and Eosin Staining Kit (Beyotime, Shanghai, China). Images were photographed using a phase contrast microscope (Leica, Germany).

The IHC scoring of human samples was performed using a modified Histo-score (H-score) by two independent pathologists as we previously described (Chen et al., 2013). The H-score was assessed as (proportion of positive cells) × (intensity of staining). The proportion of positively stained cells was scored as 0–100%. The intensity score was as the following standard: 0 (no staining), 1 (weak staining), 2 (moderate staining), and 3 (strong staining).

### Statistical Analysis

All experiments were performed at least five times using GraphPad Prism version 7.0 (GraphPad Software, San Diego, CA). All data were presented as mean ± SD. Student’s *t* test was performed to compare the differences between two groups. We compared three or more groups with one-way ANOVA with Tukey’s *post hoc* test; the overall *F* test was significant (*P* < 0.05), and there was no significant variance in homogeneity. All statistical tests were two-sided, and *P* < 0.05 was considered statistically significant.

## Results

### AZD1775 Inhibits Wee1 Activities in ESCC Cells

**Figure 1:**
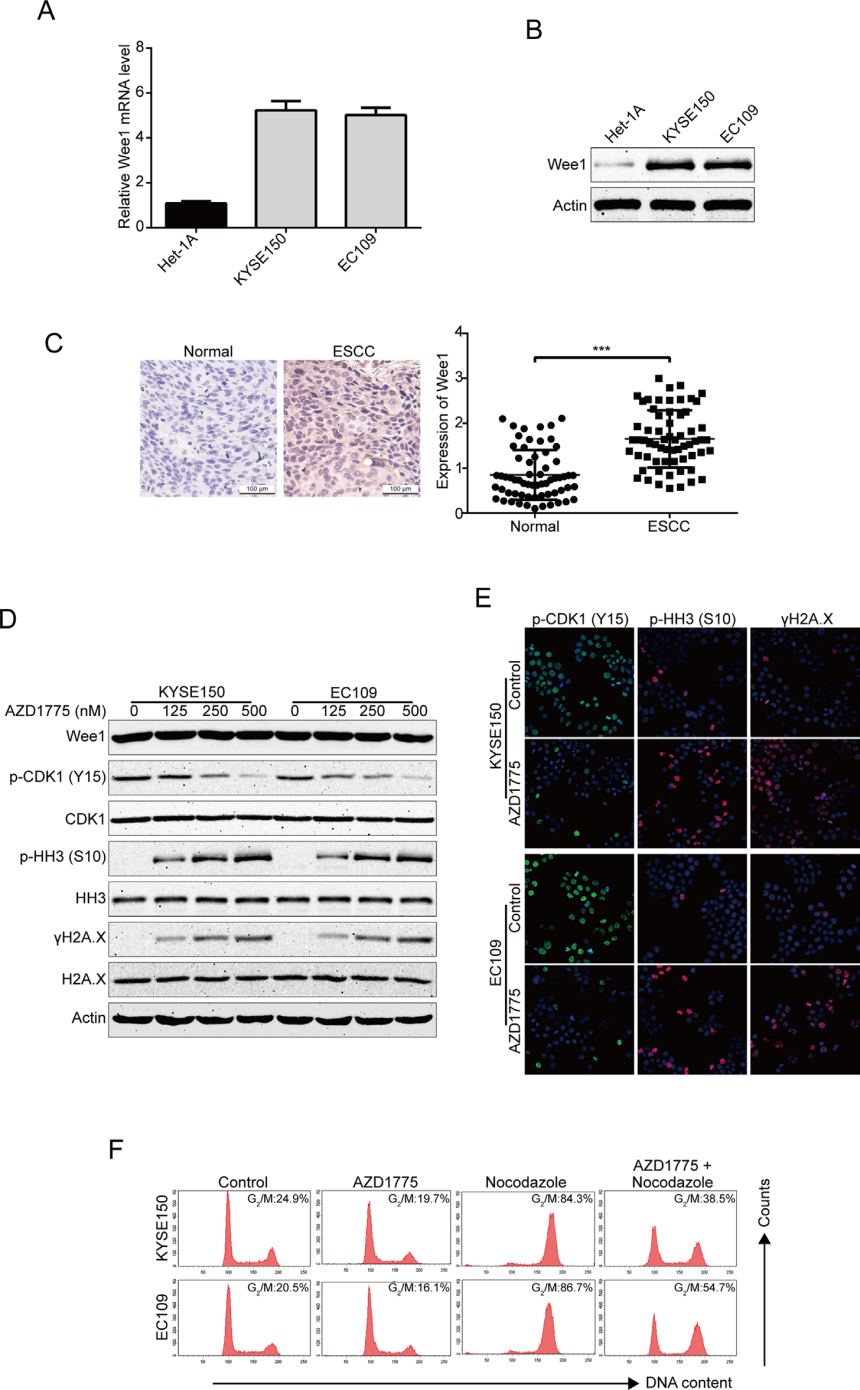
AZD1775 inhibits the activities of Wee1 kinase in ESCC cells. **(A)** qRT-PCR was used to detected the mRNA levels of Wee1 in one immortalized esophageal epithelial cell line (Het-1A) and two ESCC cell lines (KYSE150 and EC109). **(B)** The basic protein level of Wee1 were examined by Western blotting analysis. **(C)** Representative IHC micrographs (*left*) and summary bar chart (*right*) of Wee1 protein expression in ESCC tissues (63 pairs of ESCC cases and the matched adjacent normal tissues). Scale bars, 100 μm. **(D, E)** ESCC cells were treated with increasing concentrations of AZD1775 for 24 h, Western blotting **(D)** and immunofluorescence staining **(E)** were used to detect the protein levels of phospho-CDK1 (Y15), phospho-histone H3 (S10) and γH2A.X. **(F)** KYSE150 and EC109 cells were treated with or without nocodazole (10 ng/ml) for 8 h, followed by 250 nM AZD1775 treatment for another 16 h. The cells were then collected, fixed, and analyzed by flow cytometry after staining with PI. *P < 0.05 by Student’s *t*-test.

To determine the expression of Wee1 in human ESCC cells, we first detected the mRNA and protein expression of Wee1 in two ESCC cell lines (KYSE150 and EC109) and one immortalized human esophageal epithelial cell line (Het-1A) using qRT-PCR and immunoblotting analysis, respectively. As shown in **Figures 1A, B**, both the mRNA and protein levels of Wee1 were much higher in KYSE150 and EC109 cells compared to that in the control normal Het-1A cells. To further investigate the expression of Wee1 in ESCC, we performed IHC staining for Wee1 in 63 pairs of ESCC patient specimens and their corresponding adjacent normal esophageal tissue specimens. The results showed that Wee1 was much more strongly expressed in ESCC samples than those in normal esophageal tissues (**Figure 1C**). We next evaluated the inhibitory effect of AZD1775 on Wee1 kinase activity in ESCC cells by Western blotting analysis. KYSE150 and EC109 were treated with increasing concentrations of AZD1775 for 48 h. The results showed that AZD1775 dose-dependently decreased the phospho-CDK1 (Y15), a well-known downstream target of Wee1 (**Figure 1D**). Western blotting analysis also showed that AZD1775 dose-dependently promoted the expression of the phospho-Histone H3 at the S10 site (premature mitosis marker) and **γ**H2A.X (DNA damage marker), without alternating the expression of Wee1, CDK1, histone H3, and H2A.X (**Figure 1D**). We also determined the inhibitory effect of AZD1775 on Wee1 kinase activity in ESCC cells by immunofluorescence staining. The results showed that AZD1775 treatment significantly attenuated phospho-CDK1 (Y15) expression but remarkably promoted the expression of phospho-Histone H3 (S10) and γH2A.X (**Figure 1E**), which was in line with the results of Western blotting analysis (**Figure 1D**). These data indicate that AZD1775 can effectively inhibit the cellular Wee1 activity in ESCC cells.

If AZD1775 inhibited the activity of Wee1, then it was predicted to decrease the cells at the G_2_/M-phase of the cell cycle. Indeed, KYSE150 and EC109 cells largely diminished in G_2_/M-phase upon AZD1775 treatment (**Figure 1F**). As shown in **Figure 1F**, nocodazole alone treatment could block the cell cycle at G_2_/M phase compared to those cells pretreated with nocodazole for 8 h, then cotreated with AZD1775 and nocodazole for another 16 h. Although AZD1775 alone did not significantly influence cell cycle phase distribution, the combination of nocodazole and AZD1775 led to a significant decrease in G_2_/M-phase arrest for both KYSE150 and EC109 cells (**Figure 1F**). These results suggest that AZD1775 can inhibit the kinase activity of Wee1 and reduced the cell cycle at the G_2_/M-phase in ESCC cells.

### AZD1775 Inhibits the Growth of ESCC Cells

**Figure 2:**
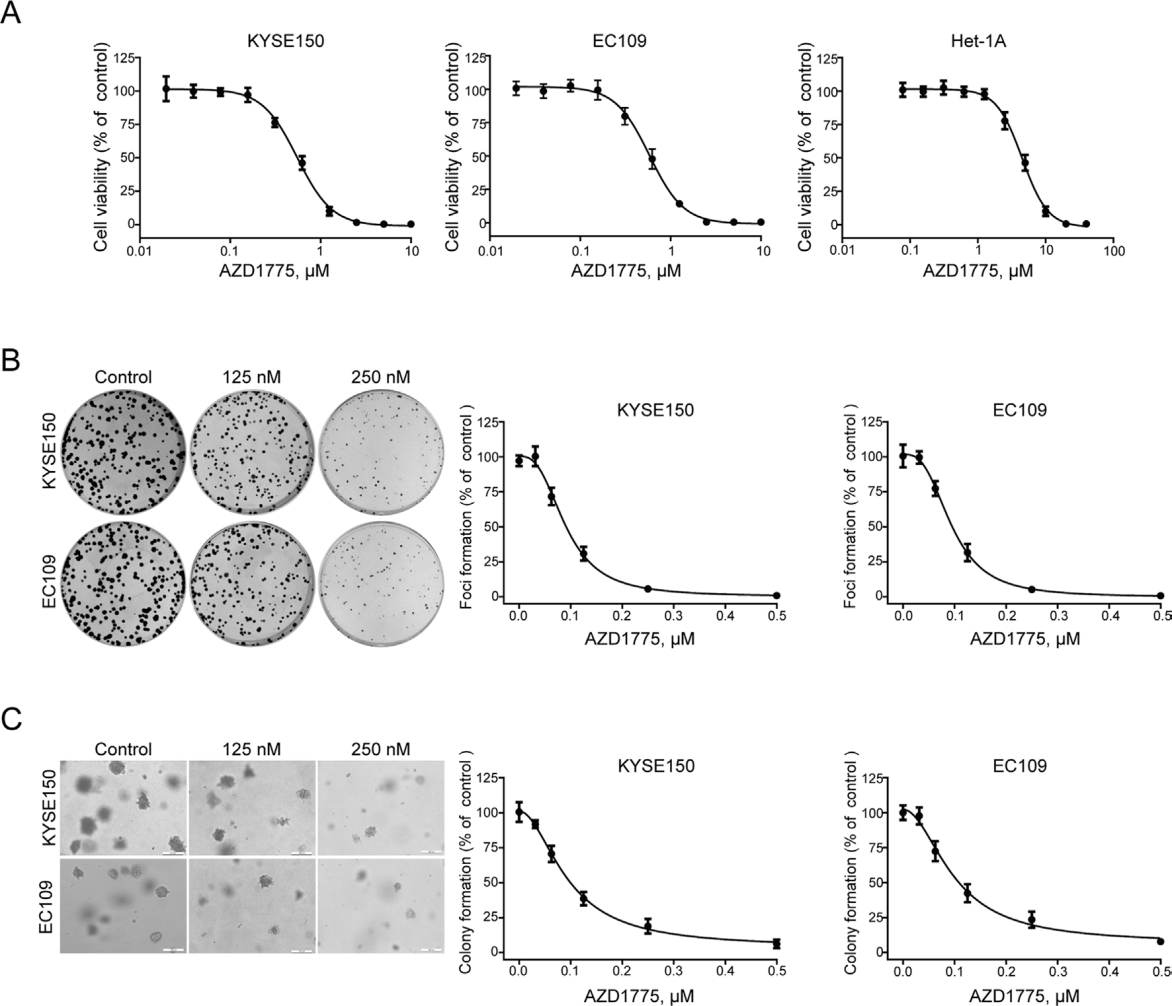
AZD1775 diminishes the growth of ESCC cells in vitro. **(A)** KYSE150 and EC109 cells were incubated with various concentrations of AZD1775 for 72 h. Cell viability was determined with MTT assay. Data from five independent experiments were shown. **(B, C)** KYSE150 and EC109 cells were exposed to increasing concentrations of AZD1775 for 48 h. The cells were then washed and subjected to focus colony formation **(B)** and soft agar assay **(C)** and incubated for 14 days, survival colonies were then counted. Data are expressed as mean ± SD, from six independent experiments.

To determine the anticancer effect of AZD1775 on ESCC cells, the MTT assay was performed to assess the proliferation of KYSE150, EC109, and Het-1A cells following AZD1775 treatment. As shown in **Figure 2A**, after incubating with AZD1775 for 72 h, the cell viability of KYSE150 and EC109 were drastically decreased in a dose-dependent manner, with the IC_50_ values of 0.55 ± 0.01 μM and 0.58 ± 0.02 μM, respectively. However, the IC_50_ value of AZD1775 in Het-1A cell was 4.51 ± 0.17 μM, suggesting that AZD1775 is significantly more cytotoxic to ESCC cells than normal esophageal cells. We next determined the effect of AZD1775 on clonogenicity in ESCC cells by focus formation assay. KYSE150 and EC109 were treated with increasing concentrations of AZD1775 for 48 h and were then subjected to colony formation frequency in the absence of drugs. AZD1775 potently diminished the number of surviving colonies of ESCC cells (**Figure 2B**). Furthermore, the results from soft agar assay, which reflects an anchorage-independent growth *in vitro*, also showed that AZD1775 dose-dependently inhibited the clonogenecity of KYSE150 and EC109 cells, with IC_50_ values of 94.6 ± 2.2 nM and 98.8 ± 4.5 nM, respectively. (**Figure 2C**). Collectively, these results demonstrate that AZD1775 has potent antiproliferation effects on ESCC cells *in vitro*.

### AZD1775 Induces Apoptosis in ESCC Cells

**Figure 3:**
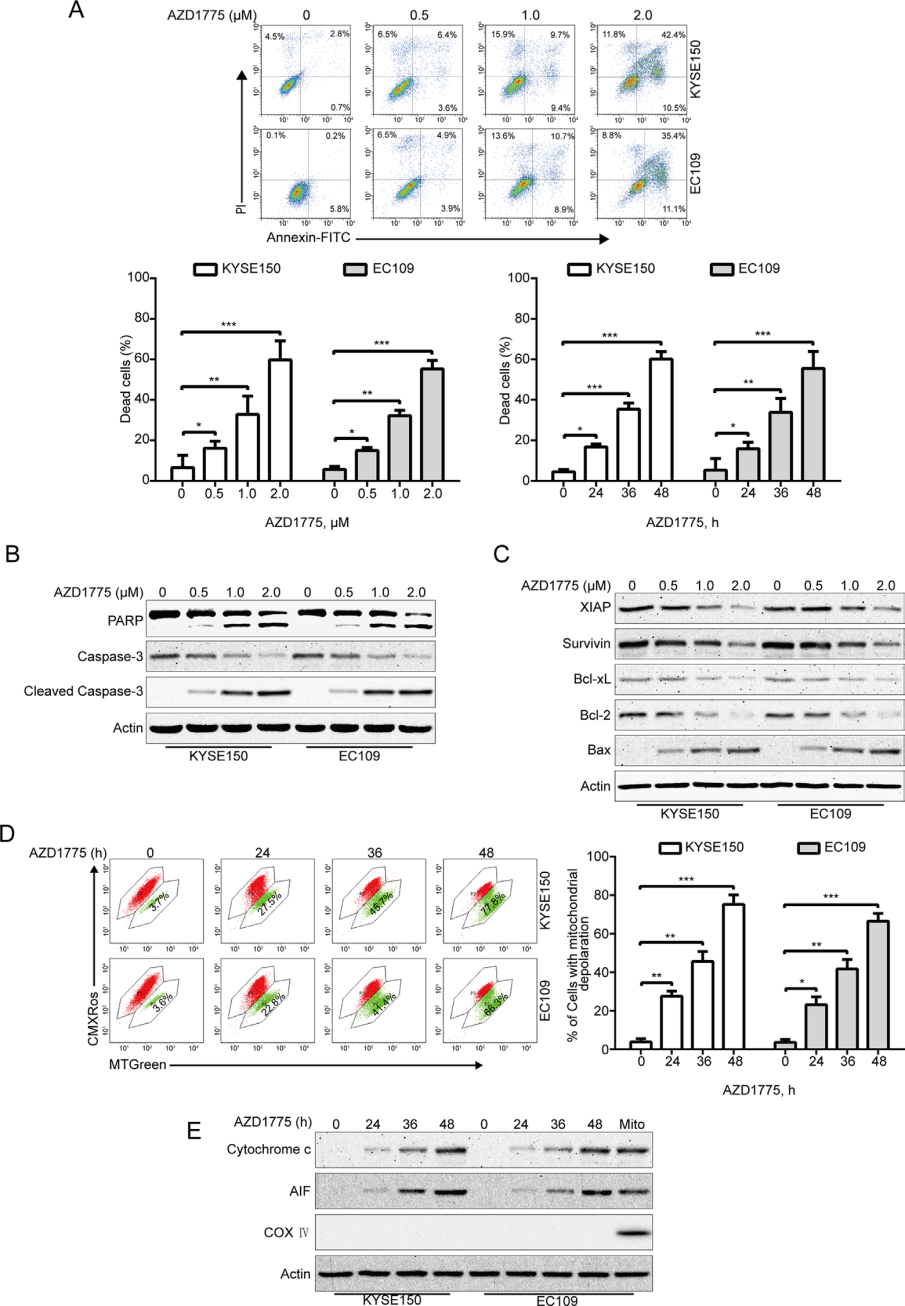
AZD1775 triggers apoptosis in ESCC cells. **(A)** ESCC cells (KYSE150 and EC109) were treated with escalating concentrations of AZD1775 for 48 h, or 2.0 μM AZD1775 for different times; cells were then subjected to flow cytometry analysis after dual staining with annexin V-FITC and PI. Upper panel: Representative histograms were shown. Lower panel: the vertical axis presents the sum of the top left and all right quadrants. Data are expressed as mean ± SD, from five independent experiments. *P < 0.05, **P < 0.01, ***P < 0.001, one-way ANOVA with *post hoc* intergroup comparison by the Tukey’s test. **(B, C)** KYSE150 and EC109 cells were incubated with various concentrations of AZD1775 for 48 h, immunoblotting analysis was performed to detect the apoptosis-related proteins. Actin served as loading control. **(D)** KYSE150 and EC109 cells treated with or without 0.5 μM AZD1775 for increasing durations, and then, the mitochondrial membrane potential was assessed by flow cytometry after CMXRos/MTGreen double staining. Right: results from five independent experiments. *P < 0.05, **P < 0.01, ***P < 0.001, one-way ANOVA with *post hoc* intergroup comparison by the Tukey’s test. **(E)** AZD1775 induced the release of cytochrome c and AIF into cytosol in ESCC cells. Levels of cytochrome c and AIF in the cytosolic extracts prepared with digitonin buffer were examined by western blotting analysis. The cytosolic fractionations were not contaminated as indicated by COX IV. Actin served as internal control.

Given that apoptosis is one of the most important forms of cell death triggered by chemotherapeutic drugs, we investigate whether AZD1775 induces ESCC cell apoptosis; we measured the apoptotic rate by flow cytometry analysis following Annexin V-FITC/PI dual staining. After AZD1775 treatment for 48 h, cells were harvested, followed by incubation with Annexin V-FITC/PI solution and then subjected to flow cytometry analysis. As shown in **Figure 3A**, AZD1775 treatment resulted in a remarkable apoptotic cell death in a dose- and time-dependent manner in both KYSE150 and EC109 cells. Western blotting results also showed that AZD1775 treatment led to a concentration-dependent increase in the cleaved PARP and active caspase-3 (two important apoptotic markers) (**Figure 3B**). To further explore the mechanisms underlying AZD1775-induced apoptosis, we next evaluated the influence of AZD1775 on the levels of several apoptosis-related proteins by immunoblotting analysis. As shown in **Figure 3C**, the expression of antiapoptotic proteins XIAP, Bcl-xL, Survivin, and Bcl-2 were greatly attenuated in a concentration-dependent fashion, whereas the protein level of proapoptotic protein Bax was upregulated. The change in these mitochondrial-related proteins prompted us to investigate the effect of AZD1775 on mitochondrial transmembrane potential. KYSE150 and EC109 cells were treated with AZD1775 for increasing times and then subjected to flow cytometry after staining with CMXRos and MTGreen. The proportion of ESCC cells with loss of mitochondrial potential was significantly increased (**Figure 3D**), which was in line with other apoptotic indices such as the cleavages of PARP and caspase-3 (**Figure 3B**). We then assessed the release of cytochrome c by Western blotting analysis. As shown in **Figure 3E**, upon treatment with AZD1775, a time-dependent upregulation of cytochrome c was observed in cytosolic fractions derived from the KYSE150 and EC109 cells. In addition, AZD1775 also induced release of AIF from mitochondria into cytoplasm (**Figure 3E**). Overall, these data indicate that AZD1775 may induce apoptosis *via* the mitochondrial (intrinsic) pathway in ESCC cells.

### AZD1775 Suppresses the Migration and Invasion of ESCC Cells

**Figure 4:**
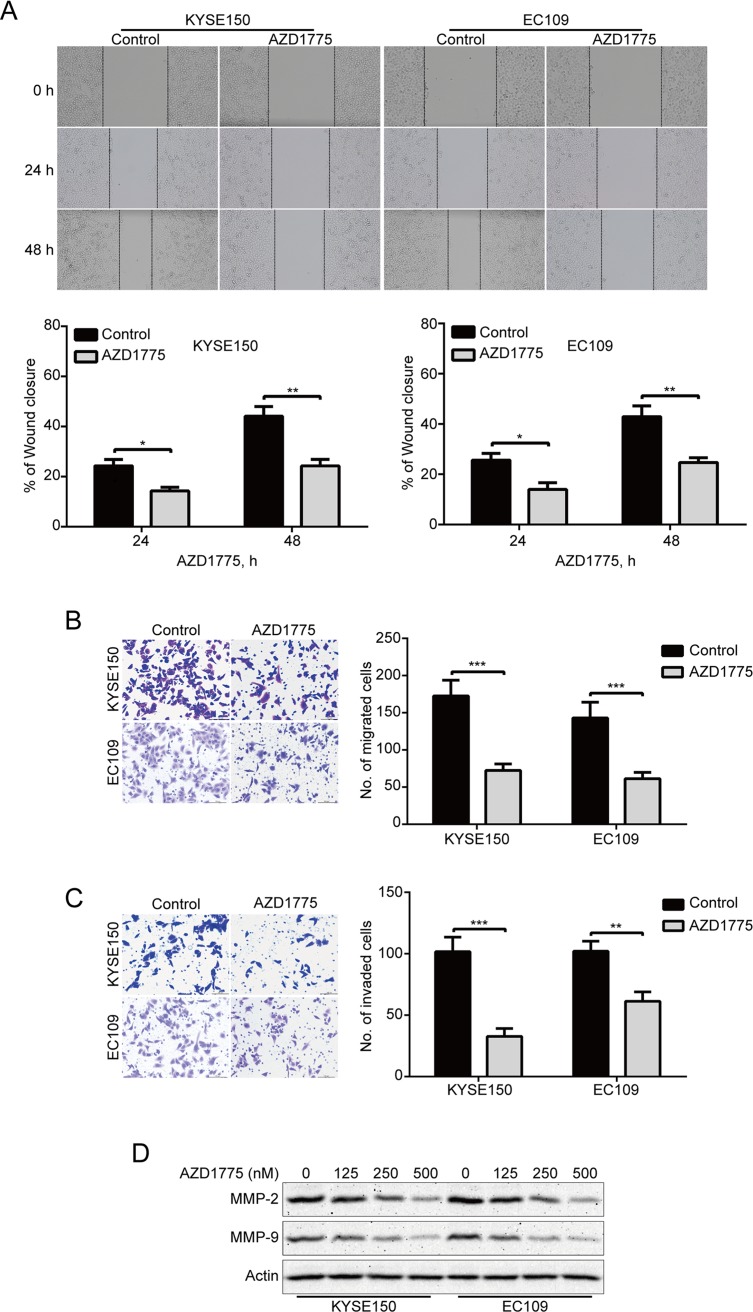
AZD1775 suppresses migration and invasion in ESCC cells. **(A)** KYSE150 and EC109 cells treated with or without 250 nM AZD1775 for 0, 24, and 48 h after scratching. The average gap width was used to evaluate migration. Bottom: quantitative analysis of the relative breadth of the wound. The wound breadth was normalized to the initial time point (0 h). Columns and error bars represent mean ± SD (*n* = 6 per group). **(B, C)** KYSE150 and EC109 cells were treated with 250 nM AZD1775 for 48 h and then subjected to transwell migration **(B)** and invasion **(C)** assay. Left: representative images; right: quantitative analysis from five random fields. Scale bar: 100 μm. Mean; error bar, SD. **(D)** Western blotting analysis of whole-cell lysates of KYSE150 and EC109 cells that were treated with different concentrations of AZD1775 for 48 h. *P < 0.05, **P < 0.01, ***P < 0.001, by Student’s *t*-test.

To determine whether AZD1775 affects the migration and invasion of ESCC cells, we first detected the ability of migration by wound healing assay. Our results showed that AZD1775 remarkably suppressed the wound healing ability of KYSE150 and EC109 cells (**Figure 4A**). Moreover, Boyden chamber transwell assay also showed that AZD1775, at the concentration of 0.25 μM, could significantly attenuate the migration ability of KYSE150 and EC109 cells (**Figure 4B**). We also performed transwell invasion assays to analyze whether AZD1775 inhibits ESCC cell invasion. As shown in **Figure 4C**, AZD1775 dramatically inhibited the invasion of KYSE150 and EC109 cells. Furthermore, upon AZD1775 treatment, the protein levels of MMP-2 and MMP-9, two critical enzymes involved in degrading the extracellular matrix, which play an important role in tumor-invasive and metastatic processes in various malignant cancers including ESCC (Li et al., 2009a; Chen and Pan, 2017), were greatly decreased (**Figure 4D**), Collectively, these data suggest that AZD1775 effectively inhibits the expression of MMP-2 and MMP-9 as well as the migration and invasion of ESCC cells.

### Wee1 shRNA Suppresses ESCC Cell Growth, Migration and Invasion

**Figure 5:**
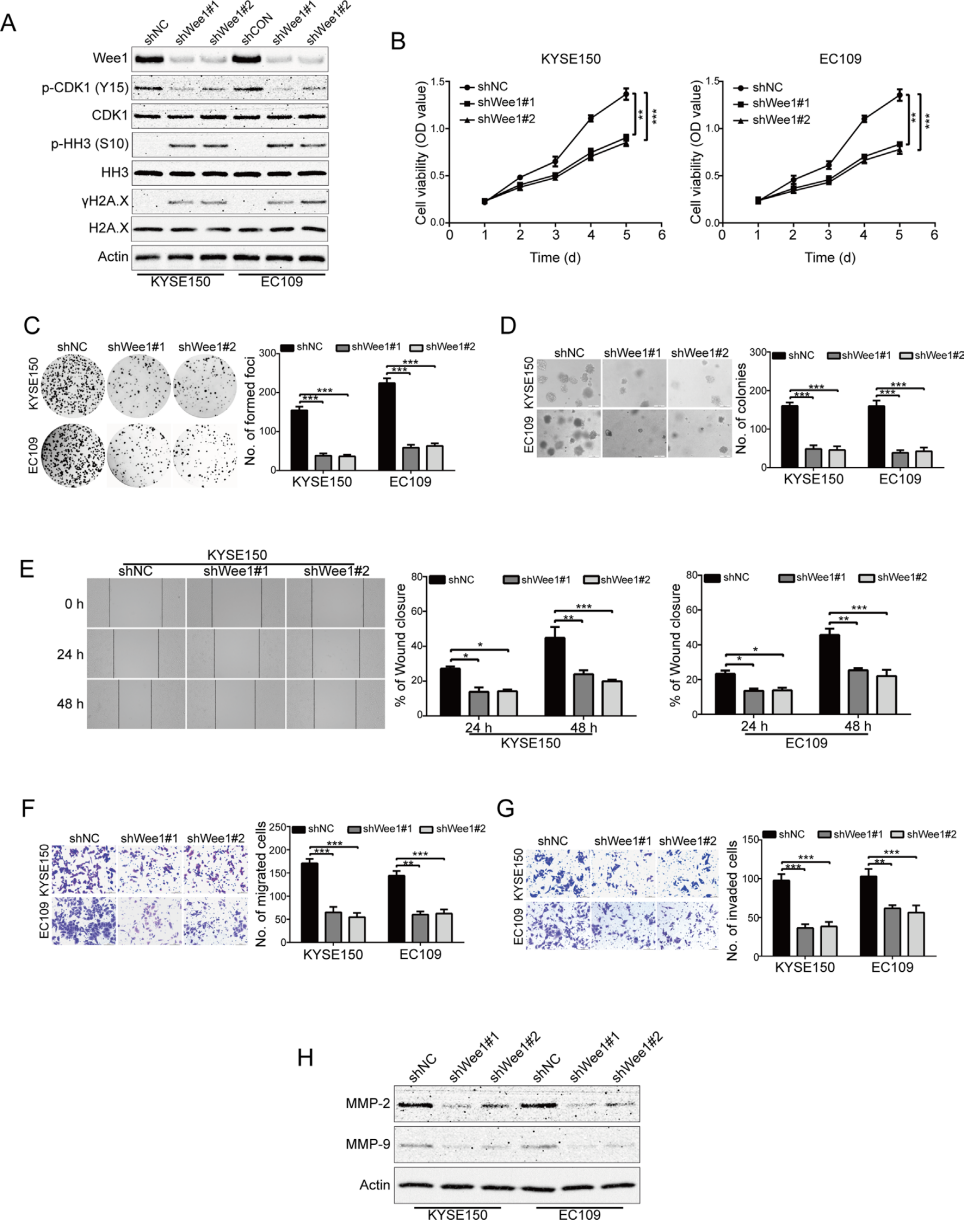
Knockdown of Wee1 inhibits proliferation, migration and invasion of ESCC cells. **(A)** Western blotting analysis of the indicated proteins in KYSE150 and EC109 cells stably transfected with the indicated shRNA. **(B–D)** MTT **(B)**, focus colony formation **(C)**, and soft agar clonogenic **(D)** assays were performed to detect the cell growth of ESCC cells that stably knocked down for Wee1 by shRNA (*n* = 6 per group). shNC: control shRNA; shWee1#1 and shWee1#2: two different shRNAs targeting Wee1. **(E–G)** Wound healing **(E)**, transwell migration **(F)**, and invasion **(G)** assays of KYSE150 and EC109 cell stable clones with knockdown of Wee1 (*n* = 6 per group). Scale bar: 100 μm. **(H)** Western blotting analysis of MMP-2 and MMP-9 expression in Wee1-knockdown ESCC cells by shRNA. Actin served as a loading control. *P < 0.05, **P < 0.01, ***P < 0.001, versus shNC; *P* values were obtained by one-way ANOVA with *post hoc* intergroup comparison with Tukey’s test.

To specifically determine the effect of Wee1 inhibition, KYSE150 and EC109 cells were stably transduced with lentiviruses encoding Wee1 shRNA (shWee1#1 and shWee1#2). Immunoblotting results showed that the protein level of Wee1 was remarkably downregulated in cells transduced with Wee1 shRNA compared to that derived from shNC (**Figure 5A**). In addition, knockdown of Wee1 by shRNA also drastically attenuated phospho-CDK1 (Y15) expression but enhanced the expression of phospho-histone H3 (S10) and γH2A.X (**Figure 5A**), which was consistent with the results upon AZD1775 treatment (**Figures 1D, E**). The MTT, colony formation, and soft agar assays showed that specific knockdown of Wee1 significantly suppressed the growth of KYSE150 and EC109 cells in comparison with those cells transduced with shNC (**Figure 5B–D**). Similarly, silencing Wee1 also significantly halted the wound-healing ability of KYSE150 and EC109 cells (**Figure 5E**). The inhibitory effect of Wee1 shRNA on the migration of ESCC cells was further demonstrated by the transwell migration assay (**Figure 5F**). Furthermore, silencing Wee1 greatly attenuated the invasive capacity of KYSE150 and EC109 cells compared to those derived from shNC (**Figure 5G**). Interestingly, the protein levels of MMP-2 and MMP-9 were lower in ESCC cells transduced with Wee1 shRNA than the control groups (**Figure 5H**). Taken together, these data suggest that, like AZD1775, specifically silencing Wee1 diminishes the aggressiveness of ESCC cells.

### AZD1775 is Synergistic With 5-FU and CDDP

**Figure 6:**
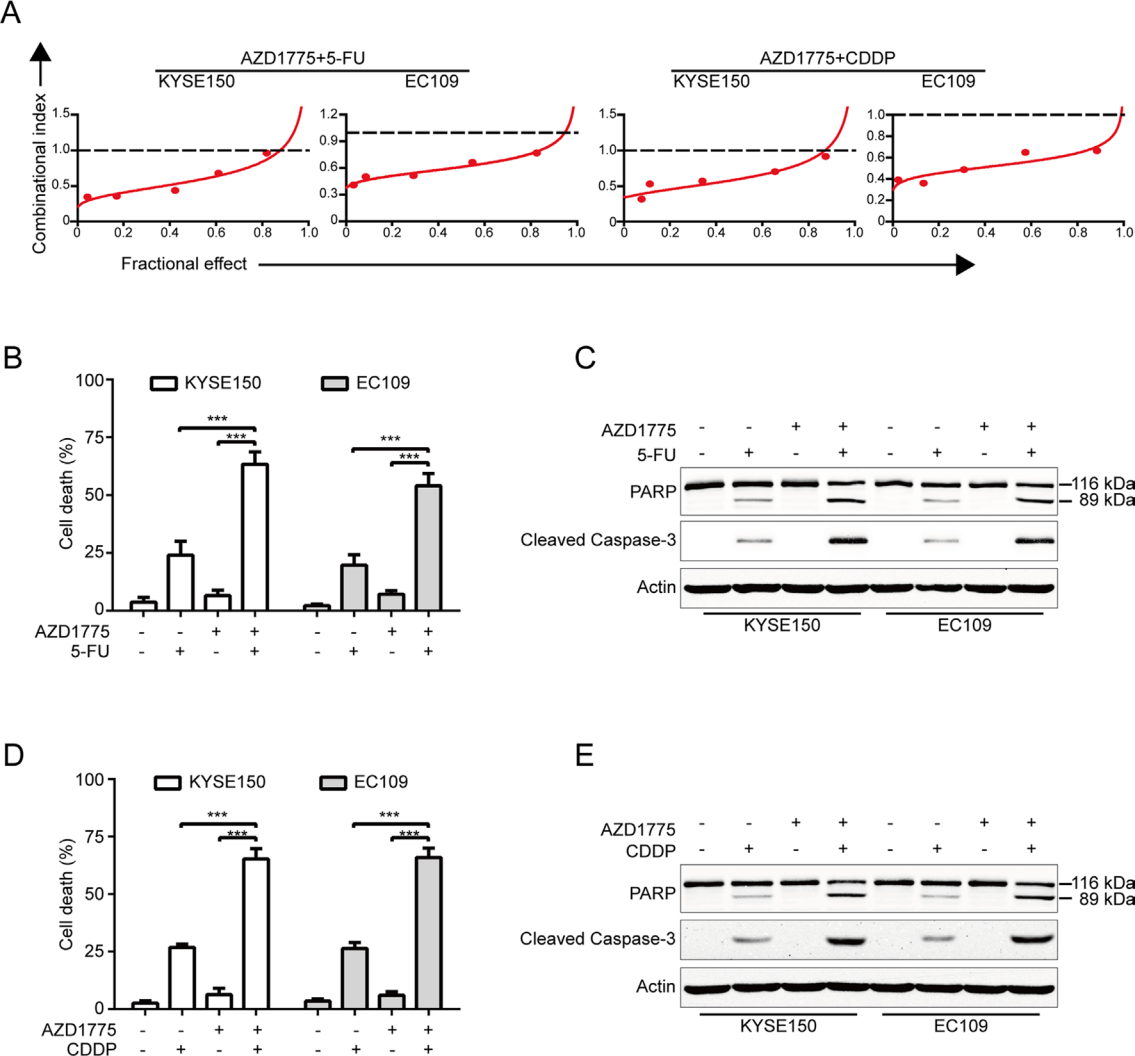
AZD1775 is Synergistic with Cisplatin and 5-FU. **(A)** KYSE150 and EC109 cells were treated with a serially diluted mixture (a fixed ratio) of AZD1775 and CDDP or 5-FU for 72 h; the cell viability was examined by MTT assay. Synergistic effect was estimated using the median-effect method of Chou and Talalay. The combination index (CI) was the ratio of the combination dose to the sum of the single-agent doses at an isoeffective level. The dashed dotted line indicates CI = 1. CI < 1 indicate synergism between the two drugs. **(B)** and **(D)** KYSE150 and EC109 cells were exposed to AZD1775 (0.25 μM) with combination of 250 μM 5-FU or 10 μM CDDP for 48 h and then examined with a hemocytometer by trypan blue exclusion assay (*n* = 6 per group). Column, mean; error bar, SD. **(C)** and **(E)** The cleavages of PARP and caspase-3 were detected by Western blotting analysis. ***P < 0.001, one-way ANOVA with post-hoc intergroup comparison with Tukey’s test.

Considering that combination between conventional chemotherapeutic agents and molecular-targeted drugs is an effective strategy to promote the survival of patients with ESCC, we then investigated whether there is a synergism between AZD1775 and the first-line chemotherapeutic drugs for ESCC including 5-FU and CDDP in inducing growth inhibition. KYSE150 and EC109 cells were incubated with increasing concentrations of AZD1775 and 5-FU or CDDP for 72 h, cells were then subjected to MTT assay, and the median-effect method of Chou and Talalay was performed to assessed the combined effect. The results showed that AZD1775 was synergistic with both drugs in suppressing the cell viability of KYSE150 and EC109 cells (**Figure 6A**). With another approach, KYSE150 and EC109 cells were treated with 0.25 μM AZD1775 in combination with 250 μM 5-FU or 10 μM CDDP for 48 h. The proportion of dead cells was assessed using trypan blue staining assay. The results showed that AZD1775 had no significant toxicity to ESCC cells; 5-FU or CDDP alone induced minimal lethality either (**Figures 6B, D**). However, combination treatment between AZD1775 and 5-FU or CDDP resulted in a significant increase in the dead cells in both KYSE150 and EC109 cells (**Figures 6B, D**). In addition, Western blotting analysis also confirmed that combination between AZD1775 and CDDP or 5-FU led to enhanced apoptosis, as presented by upregulation of cleaved PARP and activation of caspase-3 (**Figures 6C, E**), which was in line with the results of trypan blue exclusion assay (**Figures 6B, D**). Overall, these results encourage a combinational regimen of AZD1775 and 5-FU or CDDP for the treatment of patients with ESCC.

### AZD1775 Diminishes ESCC Cell Growth and Metastasis

**Figure 7:**
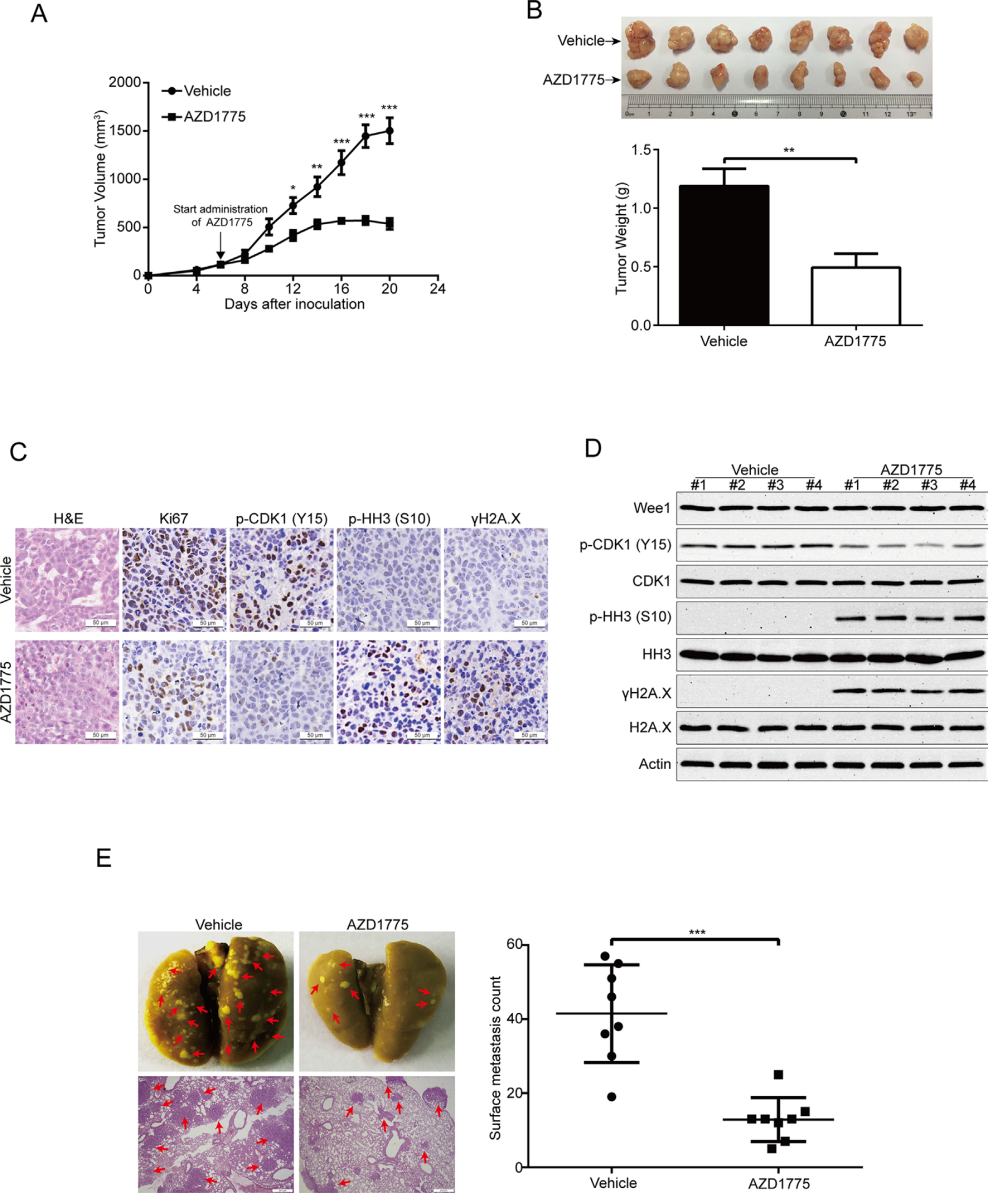
AZD1775 inhibits ESCC cell growth and metastasis in nude mice. **(A)** Nude mice bearing KYSE150 xenograft tumors were treated with vehicle (0.5% methylcellulose) or AZD1775 (60 mg/kg, treated by oral gavage daily) from day 6 to 20 after inoculation of KYSE150 cells. The tumor growth curves were shown. Point, mean; error bar, SD. *n* = 8 per group. **(B)** After 14 days of AZD1775 treatment, the mice were killed, and tumors were dissected, weighed, and photographed. Top: Representative tumors from the control and experimental group are shown; Bottom: Comparison of tumor weights in control and AZD1775 treated group (*n* = 8 per group). **(C)** H&E staining and IHC analysis of Ki67, phospho-CDK1 (p-CDK1^Y15^), phospho-histone H3 (p-HH3^S10^) and γH2A.X in xenograft tissues from mice. Scale bar: 50 μm. **(D)** Immunoblotting of the Wee1, p-HH3^S10^, HH3, p-CDK1^Y15^, CDK1, γH2A.X, and H2A.X proteins in xenograft tissues was shown. **(E)** KYSE150 cells were intravenously injected into nude mice *via* the lateral tail vein. Left: Representative images of lungs (upper panel) and lung sections stained with H&E (lower panel) harvested 8 weeks postinjection (arrows indicate the metastatic nodules). Right: Surface metastatic nodules in the lungs were counted. Data represent mean ± SD (*n* = 8 per group). Scale bar, 200 μm. *P < 0.05, **P < 0.01, ***P < 0.001, by Student’s *t*-test.

To further evaluate the antitumor effects of AZD1775 on ESCC, we subcutaneously inoculated KYSE150 cells in BALB/c nude mice. Six days later, when the tumor volume of xenografts was ∼100 mm^3^, the mice were randomly separated into two groups (eight mice per group): AZD1775 (60 mg/kg) or vehicle (0.5% methylcellulose) for 14 days. The tumor growth curve (time curve versus tumor volume) of KYSE150 tumors was remarkably attenuated by AZD1775 (**Figure 7A**). Moreover, the tumor weight in the vehicle-treated mice was significantly higher than the AZD1775-treated mice (**Figure 7B**). The IHC staining results also showed that the expression of proliferation marker Ki67 was greatly attenuated by AZD1775 (**Figure 7C**). In addition, the protein level of phospho-CDK1 (Y15) was substantially attenuated in the AZD1775-treated group, which indicated that AZD1775 could inhibit the kinase activity of Wee1 *in vivo* (**Figure 7C**). Moreover, tumors in the AZD1775-treated animals showed increased expression of phospho-histone H3 (Ser 10) and γH2A.X compared to those from the vehicle-treated mice (**Figure 7C**). Consistently, Western blotting results showed that AZD1775 drastically inhibited the p-CDK1 (Y15) compared with that of control mice (**Figure 7C**). Interestingly, the expression of p-histone H3 (Ser 10) and γH2A.X was dramatically upregulated, while the total amount of Wee1, CDK1, HH3, and H2A.X protein were not alternated (**Figure 7D**).

Given that Wee1 inhibition by AZD1775 significantly inhibited the migration and invasion of ESCC cells *in vitro*, we then explored whether AZD1775 suppresses the metastasis of ESCC cells *in vivo*. As shown in **Figure 7E**, AZD1775 significantly attenuated the metastatic ability of ESCC cells in comparison with the vehicle-treated mice, as demonstrated by the decreased number in metastatic tumor nodules in the lungs of the AZD1775-treated mice (**Figure 7E**). Furthermore, the H&E staining analysis also showed an obviously decrease in number and size of the metastatic nudes in the lungs derived from AZD1775-treated mice compared to those of the vehicle treatment (**Figure 7E**).

Taken together, these findings further demonstrate that AZD1775 can effectively suppress ESCC cell growth and metastasis *in vivo*.

## Discussion

In the present study, we showed that Wee1 was overexpressed in ESCC cell lines and clinical samples. More importantly, we analyzed the inhibitory effect of the Wee1 inhibitor AZD1775 on ESCC cells both *in vitro* and *in vivo*. Interestingly, we found that AZD1775 significantly suppressed the growth, migration, and invasion of ESCC cells. The expression of MMP-2 and MMP-9 was simultaneously downregulated. In addition, knocking down Wee1 by shRNA phenocopied the inhibitory effects of AZD1775 on ESCC cell proliferation, colony formation, migration, and invasion. AZD1775 also induced cell apoptosis *via* the mitochondrial-dependent signaling pathway and had a synergistic inhibitory effect on cell growth when combined with 5-FU or CDDP. Furthermore, AZD1775 could remarkably attenuate the growth and lung metastasis of ESCC cells in nude mice. To our knowledge, this is the first report to detect the expression of Wee1 in ESCC and to elucidate the antitumor effect of Wee1 inhibitor AZD1775 on ESCC cells, using both *in vitro* and *in vivo* models.

AZD1775 is first in class, highly selective, and effectively suppresses the kinase activity of recombinant human Wee1 (IC_50_ = 5 nM) by competing with ATP-binding site (Hirai et al., 2009). The antiproliferative roles of AZD1775 have been found in a large number of preclinical studies (Pfister et al., 2015; Richer et al., 2017; Webster et al., 2017). The mechanism of AZD1775-mediated cell growth inhibition has been largely attributed to either DNA damage response or premature mitosis (Do et al., 2015; Lewis et al., 2017; Webster et al., 2017). Consistent with these findings, in the present study, we demonstrated that AZD1775 effectively suppressed the Wee1 kinase, with direct evidence of decrease in the protein level of phospho-CDK1 (Y15). In addition, the expression of γH2A.X and phospho-histone H3 (S10) were drastically enhanced upon AZD1775 treatment. The MTT, colony formation, and soft agar assays also confirmed that AZD1775 at low nanomolar concentrations potently inhibited the growth of ESCC cells. More importantly, our *in vivo* data also demonstrated that AZD1775, at an oral dose of 60 mg/kg/day, greatly attenuated the tumor growth of xenografted KYSE150 cells and significantly suppressed the phospho-CDK1 (Y15) expression, indicating that AZD1775 might be a potential therapeutic drug for the treatment of ESCC.

Increasing evidence showed that AZD1775 can induce significant apoptosis in various cancers including sarcomas (Kreahling et al., 2012), glioblastoma (Lescarbeau et al., 2016), lung cancer (Chen et al., 2017), and gastric cancer (Kim et al., 2016). In agreement with these findings, in our study, we observed that AZD1775 treatment indeed led to an increase in cell apoptosis in ESCC cells, as demonstrated by a dose- and time-dependent increase in annexin-V-binding cells and upregulation of cleaved PARP and caspase-3. More importantly, we also demonstrated that the AZD1775-mediated ESCC cell apoptosis was *via* the mitochondrial-dependent pathway because AZD1775 caused a decrease in mitochondrial transmembrane potential and triggered the release of cytochrome c and AIF into the cytoplasm. Moreover, the levels of antiapoptotic proteins, including Survivin, XIAP, Bcl-2, and Bcl-xL, were decreased, but the protein level of proapoptotic Bax was significantly overexpressed in AZD1775-mediated apoptotic ESCC cells. Future work is needed to further define the precise mechanism underlying the proapoptotic effect of AZD1775.

Tumor metastasis is one of the main reasons that leads to ESCC patients with poor prognosis (Li et al., 2009a). In this study, we found that targeting Wee1 by shRNA or AZD1775 significantly diminished the migration and invasion and suppressed the protein expression of MMP-2 and MMP-9 in ESCC cells. More importantly, AZD1775 also drastically suppressed the lung metastasis in nude mice. Consistent with our results, a previous report showed that knockdown of Wee1 by siRNA decreases the migration and invasion of gastric cancer cells (Kim et al., 2016). In addition, Bukhari *et al.* recently reported that a combination of bioavailable AZD1775 and ATR inhibitor AZD6738 strongly suppressed metastasis with minimal side effects in an orthotopic breast cancer model (Bukhari et al., 2019). These findings suggest that AZD1775 alone or in association with other regents could be a promising therapeutic strategy for metastatic ESCC patients.

It is well known that combination approaches to therapy are helpful for enhancing the efficiency of molecular-targeted agents in the treatment of aggressive solid tumors including ESCC (Kwak et al., 2007; Sugase et al., 2017; de Castro Junior et al., 2018; Wu et al., 2018). There are amounting preclinical data about the synergistic antitumor effect between AZD1775 and targeted agents including AURKA inhibitor alisertib, PARP inhibitor olaparib, Sirt1 inhibitor Ex527, and pan-histone deacetylase inhibitor panobinostat in various malignancies (Wang et al., 2015; Chen et al., 2017; Lallo et al., 2018; Lee et al., 2019). Moreover, AZD1775, when used in combination with DNA damaging agents including carboplatin, gemcitabine, and cisplatin, has shown promising antitumor activity along with an acceptable toxicity profile in patients with advanced solid tumors (Leijen et al., 2016a; Leijen et al., 2016b). Consistent with these findings, our findings showed that there was a notable synergistic inhibitory effect between AZD1775 and 5-FU or CDDP on the growth of ESCC cells. Moreover, AZD1775 significantly enhanced the cell apoptosis triggered by these two cytotoxic agents in ESCC cells. Taken together, inhibition of Wee1 by AZD1775 in combination with either targeted drugs or cytotoxic chemotherapy might be a novel therapeutic strategy for patients with ESCC.

Previous studies showed that the antitumor effects of AZD1775 are limited to p53-deficient cancer cells, in particular when combined with other agents (Hirai et al., 2009; Hirai et al., 2010; Rajeshkumar et al., 2011). The proposed rationale is that cancer cells with a dysfunctional p53, a key component of the G_1_/S DNA-damage checkpoint, are more dependent on G_2_/M checkpoint to repair DNA damages (Matheson et al., 2016). Thus, p53 mutation has been used as a predictor to estimate whether the cancer cells are sensitive to Wee1 inhibitors in clinical trials (Indovina and Giordano, 2010; De Witt Hamer et al., 2011). However, in this study, we found that both the wild-type p53 EC109 cells (Li et al., 2012) and the mutant p53 KYSE150 cells (Komatsu et al., 2009) displayed a similar sensitivity to AZD1775 or Wee1 shRNAs. Consistent with our results, Mir SE *et al.* demonstrated that inhibition of Wee1 by a small molecule inhibitor or siRNA triggers cell death in several glioblastoma cell lines and primary cultures and did not correlate with p53 status (Mir et al., 2010). Kreahling JM *et al.* also found that AZD1775 is effective as monotherapy in sarcoma cells independent of p53 status, as evidenced by a similar degree of cell death in sarcoma cells with wild-type p53, mutant p53, and null p53 (Kreahling et al., 2012). In addition, AZD1775 sensitizes acute myelogenous leukemia to cytarabine, medulloblastoma cell lines to cisplatin, and high-grade glioma cell lines to irradiation, independently of p53 functionality (Porter et al., 2012; Harris et al., 2014; Mueller et al., 2014). Recently, Hauge S et al. reported that the p21 (a downstream target of p53)-deficient cancer cells are more sensitive to AZD1775 alone or in combination with ionizing radiation (Hauge et al., 2019), whereas both cell lines used in this study have been demonstrated to express p21 (Lin et al., 2017; Liu et al., 2018). Collectively, these findings suggest that whether the status of p53 or p21 is predictive of AZD1775 response largely depends on the types of cancer.

In summary, our findings showed that Wee1 was overexpressed in ESCC. Inhibition of Wee1 by AZD1775 effectively inhibited cell growth and induced apoptosis *via* the mitochondrial-dependent signaling in ESCC cells. AZD1775 also diminished ESCC cell migration and invasion and suppressed the lung metastasis in nude mice. Our results suggest that AZD1775 warrants further investigations to evaluate its efficacy for the treatment of ESCC patients, even in those with metastasis.

## Data Availability

The raw data supporting the conclusions of this manuscript will be made available by the authors, without undue reservation, to any qualified researcher.

## Ethics Statement

Human samples were obtained from the First Affiliated Hospital of Henan University. The research was approved by the Committees for Ethical Review of Research Involving Human Subjects in the First Affiliated Hospital of Henan University. Written informed consent was obtained from all patients prior to the study. Animal experiments were approved by the Ethics Committee of Henan University, Kaifeng, China.

## Author Contributions

SB performed the experiments. QW also complementally conducted some experiments. ZZ analyzed the data. LC perfected the experiments, prepared the figures, and wrote the manuscript. CW and SX coordinated the project and perfected the experiments. All authors read and approved the final manuscript.

## Funding

This work was supported by funding from the Postdoctoral Research Sponsorship of Luohe Medical College (No. PR20180001), the National Natural Science Foundation of China (Nos. 81772832 and 81573465), and the Program for Innovative Research Team (in Science and Technology) in University of Henan Province (No. 19IRTSTHN004).

## Conflict of Interest Statement

The authors declare that the research was conducted in the absence of any commercial or financial relationships that could be construed as a potential conflict of interest.
