# Supplementary material for: Wee1 Inhibitor AZD1775 Effectively Inhibits the Malignant Phenotypes of Esophageal Squamous Cell Carcinoma In Vitro and In Vivo
Source: Front Pharmacol. 2019 Aug 2;10:864. doi: 10.3389/fphar.2019.00864 (PMC6688135; doi:10.3389/fphar.2019.00864)
Supplement: Supplementary file 1 [file DataSheet_1.pdf]

Supplementary Table S1.

Table S1. Primers used for qRT-PCR.

Gene	Forward	Reverse
**Wee1**	5’-GCTTGCCCTCACAGTGGTATG-3’	5’-CCGAGGTAATCTACCCTGTCT-3’
**GAPDH**	5'- GAAGGTGAAGGTCGGAGTC-3'	5'- GAAGATGGTGATGGGATTTC-3'
