# Supplementary material for: Wee1 Inhibitor AZD1775 Effectively Inhibits the Malignant Phenotypes of Esophageal Squamous Cell Carcinoma In Vitro and In Vivo
Source: Front Pharmacol. 2019 Aug 2;10:864. doi: 10.3389/fphar.2019.00864 (PMC6688135; doi:10.3389/fphar.2019.00864)
Supplement: Supplementary file 2 [file DataSheet_2.pdf]

Supplementary Table S2.

Table S2. Primers used for shRNA.

Gene	Sequence (5' to 3')
**shNC (No target shRNA)**	CCGGGCGCGATAGCGCTAATAATTTCTCGAGAAATTATTAGCGCTATCGCGCTTTTT
**shWee1#1**	CCGGTAATAGAACATCTCGACTTATCTCGAGATAAGTCGAGATGTTCTATTATTTTTG
**shWee1#2**	CCGGGTGGGCAGAAGATGATCATATCTCGAGATATGATCATCTTCTGCCCACTTTTTG
